# Membrane Transporters and Carriers in Human Seminal Vesicles

**DOI:** 10.3390/jcm11082213

**Published:** 2022-04-15

**Authors:** Damian Malinowski, Paweł Grzegółkowski, Katarzyna Piotrowska, Marcin Słojewski, Marek Droździk

**Affiliations:** 1 Department of Pharmacokinetics and Therapeutic Drug Monitoring, Pomeranian Medical University, 70-111 Szczecin, Poland; damian.malinowski@pum.edu.pl; 2 Department of Urology, Pomeranian Medical University, 70-111 Szczecin, Poland; pawel.grzegolkowski@gmail.com (P.G.); marcin.slojewski@pum.edu.pl (M.S.); 3 Department of Physiology, Pomeranian Medical University, 70-111 Szczecin, Poland; piot.kata@gmail.com; 4 Department of Experimental and Clinical Pharmacology, Pomeranian Medical University, 70-111 Szczecin, Poland

**Keywords:** seminal vesicles, drug transporters, solute-linked carrier, ABC transporters

## Abstract

Seminal vesicles play an important role in the male reproductive system, producing seminal fluid and thus adequate environment for sperm. However, mechanisms underlying secretory functions of the seminal vesicles’ epithelium have not been defined yet. The aim of the present study was to characterize expression and immunolocalization of selected membrane transporters and carriers in the seminal vesicles. The study included biopsy specimens collected from non-affected parts of seminal vesicles from 53 patients of Caucasian origin subjected for prostatectomy. RT-PCR was used to define expression of 15 genes coding for ABC-family and 37 genes encoding 37 SLC-family transporters/carriers. Immunohistochemistry was used to define localization of 6 transporters. In the seminal vesicles, the following membrane transporters and carriers were defined: ABCA1, ABCB1, ABCB5, ABCB6, ABCC1, ABCC2, ABCC3, ABCC4, ABCC5, ABCC6, ABCG2, SLC01C1, SLC02B1, SLC04A1, SLC04C1, SLC10A1, SLC15A1, SLC15A2, SLC16A1, SLC16A3, SLC19A1, SLC22A1, SLC22A3, SLC22A11, SLC22A18, SLC22A4, SLC22A5, SLC28A1, SLC2A9, SLC33A1, SLC47A1, SLC47A2, SLC51A, SLC51B, SLC7A5, SLC7A6. Age-dependent expression was evidenced for ABCB1, ABCG2, SLC04C1, SLC15A1, SLC16A1, SLC22A11, SLC22A18, SLC47A1 and SLC47A2. ABCG2, P-gp, MRP1, MRP3, MCT1 and LAT1 were localized in the apical membrane and P-gp in the basolateral membrane of the seminal vesicle epithelium. The expression of the membrane transporters and carriers in the seminal vesicle epithelium confirms its secretory and barrier functions.

## 1. Introduction

Seminal vesicles constitute an integral part of the male reproductive system, producing seminal fluid and thus adequate environment for sperm. The fluid is responsible for nutrition, normal sperm cells motility, semen liquidity, stabilization of sperm cells chromatin, as well as immune modulation of the female reproductive system. All of the processes are detrimental for preservation of male fertility, and dysfunction of the seminal vesicles is associated with male infertility [1,2,3].

The epithelium of the seminal vesicles not only forms a mechanical barrier protecting sperm but also provides active defense mechanisms (transporters and carriers) as well as produces seminal fluid. In the latter ions and electrolytes (e.g., potassium, magnesium, zinc, bicarbonate, glutathione), low molecular weight substances (e.g., ascorbic acid, fructose, phospholipids, prostaglandins), structural and transport proteins (semenogelin, lactoferrin, transferrin), hormones (prolactin, testosterone, insulin), enzymes (superoxide dismutase, catalase, glutathione peroxidase, carbonic anhydrase) and enzyme protein inhibitors (sperm motility inhibitor) can be found [4,5]. Normal function of seminal vesicles is possible due to, among other factors, the presence of characteristic transporter and carrier proteins.

Membrane transporters enable relocation of endogenous and exogenous compounds through cell membranes within an effector tissue, thereby regulating cell homeostasis process and composition of extracellular fluid. Each transporter has a specific pattern of tissue expression. The membrane transporters and carriers are classified into two superfamilies, i.e., ABC (ATP-binding cassette) transporters and SLC (solute-linked carrier) carriers. ABC transporters provide efflux functions, and export molecules (substrates) out of cells using ATP as driving energy. The 48 ABC transporters are divided into seven families (ABCA to ABCG) based on their gene structure, amino acid sequence, domain organization, and phylogenetic analysis [6]. The most important are P-glycoprotein (P-gp/MDR1/*ABCB1*), multidrug-resistance-associated proteins (MRPs/*ABCC*s), and breast-cancer-resistance protein (BCRP/*ABCG2*). These transporters are considered as key regulators of intracellular accumulation of lipids, sterols, drugs and toxins. More than 400 transport proteins of SLC superfamily have been identified and are allocated to over 60 families. The classification of SLC transporter superfamily is based exclusively on biological function and not on sequence homology. Members of the same SLC transporter family demonstrate an at least 20% protein sequence identity, but share similar substrates. The SLC family mainly includes organic anion-transporting polypeptides (OATPs/*SLCO*s), organic anion transporters (OATs/*SLC22A*s), organic cation transporters (OCTs/*SLC22A*s), organic cation and carnitine transporters (OCTNs/*SLC22A*s), peptide transporters (PEPTs/*SLC15A*s) and multidrug and toxin extrusions (MATEs/*SLC47A*s). Most of SLC transporters function as influx carriers, mediating movement of solutes, either by passive diffusion along concentration gradient, by cotransport (acting as facilitated transporters), or counter-transport against concentration gradient using the concentration gradient created by another solute (acting as secondary active transporters). A class of MATEs transporters provide efflux activity. SLC proteins transport a wide array of molecules, including carbohydrates, amino acids, vitamins, nucleotides, metals, inorganic ions, organic anions, oligopeptides and drugs [7].

The ABC and SLC transporters play a very important role in cells, tissues and organ homeostasis, but their expression in seminal vesicles have not been defined yet. Herein, mRNA expression levels of membrane transporters from both ABC and SLC superfamilies with immunohistochemistry confirmation of the most expressed transporters in the seminal vesicles was determined in the present study.

## 2. Materials and Methods

### 2.1. Study Subjects

The study included 53 patients of Caucasian origin aged 61.7 ± 6.6 (from 48 to 78) years with prostate cancer (Supplementary Table S1). Biopsy specimens were collected from non-affected parts of seminal vesicles, within 5 min from dissection, during standard laparoscopic prostatectomy. In the studied cohort, coexisting chronic diseases were found in 42 (70%) patients, i.e., hypertension (58.3%) (mostly treated to angiotensin-convertase enzyme inhibitors), type 2 diabetes (15%) (mostly medicated with metformin), hypothyroidism (8.3%) (mostly supplemented with L-thyroxin), gout (5%) (mostly administered allopurinol). During the surgery, standard anesthesia was implemented, i.e., propofol, sevoflurane, rocuronium, fentanyl, metamizole. The samples were snap-frozen in liquid nitrogen (stored at −80 °C till RNA extraction), placed in formalin solution (for further immunohistochemistry analysis, 4% paraformaldehyde solution) and in RNAlater™ stabilization solution (Invitrogen™, AM7021, Waltham, MA, USA). All patients were hospitalized in Department of Urology, Pomeranian Medical University, Szczecin, Poland. The study protocol was approved by the local Bioethics Committee of the Pomeranian Medical University in Szczecin (KB-0012/152/17), the study participants gave a written informed consent.

### 2.2. mRNA Expression

Total RNA from seminal vesicle samples was isolated using Direct-zol™ RNA MiniPrep Plus (Zymo Research, R2070, Irvine, CA, USA) following the manufacturer’s procedure (approximately 20–30 mg of each tissue sample). RNA concentration and quality were assessed using DeNovix DS11 FX+ spectrophotometer (Wilmington, DE, USA). RNA samples were stored at −80 °C till further analysis. cDNA transcription was acquired using SuperScript™ IV VILO™ reverse transcription kit (Invitrogen™, 11766050, Waltham, MA, USA), according to the manufacturer’s protocol. Quantitative real-time PCR was performed using individual gene expression assays for mRNA expression analysis using ViiA7 Real-Time PCR System (Applied Biosystems, Waltham, MA, USA). Pre-validated TaqMan Gene Expression Assays were used for determining the transporter genes (Applied Biosystems, 4331182, Waltham, MA, USA) (Supplementary Table S2). Each sample was analyzed in two technical replications and mean cycle threshold (CT) values were used for further analysis. The relative constitutive gene expression was calculated by 2^−ΔCT^ method with the house-keeping genes.

### 2.3. Immunohistochemistry

Sections of seminal vesicles (4 μm thick) were hydrated, and heat-mediated antigen retrieval was performed in microwave oven in citrate buffer pH = 6 (Vector Antigen Unmasking Solution, Citric Acid Based, H-3300, Vector Labs, Burlingame, CA, USA). After cooling to room temperature (RT), the activity of peroxidase was blocked with BLOXALL Endogenous Enzyme Blocking Solution (ImmPRESS^®^ HRP Universal (Horse Anti-Mouse/Rabbit IgG), Vector Laboratories, Burlingame, CA, USA) PLUS Polymer Kit, Peroxidase, Vector Laboratories, USA cat no MP-7800). After incubation with BLOXALL, samples were washed twice with PBS (Phosphate-buffered saline) and further incubated with 2.5% Normal Horse Serum (ImmPRESS^®^ HRP Universal PLUS Polymer Kit, Peroxidase, Vector Laboratories, Burlingame, CA, USA). Further, slides were incubated with primary monoclonal antibodies: anti-ABCG2 (BPX-21 dilution 1:150) (Sc-58222, SantaCruz Biotechnology, Dallas, TX, USA), anti-LAT1 dilution 1:150 (Sc-374232, SantaCruz Biotechnology, Dallas, TX, USA), anti-MRP3 dilution 1:150 (Sc-59612, SantaCruz Biotechnology, Dallas, TX, USA), anti-MCT-1 dilution 1:100 (Sc-365501, SantaCruz Biotechnology, Dallas, TX, USA), anti-MRP-1 dilution 1:100 (Ab24102, Abcam, Cambridge, UK) and anti-MDR1 dilution 1:40 (MAB4120, Merck, Darmstadt, Germany) for 1 h in RT. A single section for each antibody for each patient was incubated with PBS instead of primary antibody for the control of primary antibodies (negative controls). After double wash in PBS slides were incubated with ImmPRESS Universal Antibody Polymer Reagent (ImmPRESS^®^ HRP Universal PLUS Polymer Kit, Peroxidase, Vector Laboratories, Burlingame, CA, USA), after washing in PBS reaction was visualized with ImmPACT DAB EqV Substrate (ImmPRESS^®^ HRP Universal PLUS Polymer Kit, Peroxidase, Vector Laboratories, Burlingame, CA, USA). After visualization slides were counterstained with hematoxilin (Harris modified Hematoxilin, Sigma, HHS32-1L, Burlington, MA, USA) and after dehydratation with ascending alcohols (70%, 96%, 99.9%) and clearing in xylene (all purchased from Sigma-Aldrich, Burlington, MA, USA), samples were mounted in Histokitt mounting medium (Karl Hecht, 41025010, Sondheim vor der Rhön, Germany). After drying in air, sections were evaluated under an Olympus IX81 inverted microscope (Olympus, Hamburg, Germany). Images were captured with ×20 objective, bright field. Yellow-to-brown pigmentation was considered positive IHC reaction and described as: -no reaction (negative), −/+ weak reaction, + positive reaction, ++ strong positive reaction, +++ very strong positive reaction. Micrographs were collected with CellSens software (Olympus, Hamburg, Germany). Luminal and basolateral membranes in epithelial cells were not distinguished. Instead, distinguishing between basal and glandular cells was performed, where glandular cells are described as cells with secretory droplet in the cytoplasm and are well distinguishable from basal (usually flat) and small cells in seminal vesicles’ epithelium.

### 2.4. Statistical Analysis

mRNA expression data are presented as mean ± standard deviation (SD). Statistical analysis was performed using STATISTICA PL, ver. 13.1 [StatSoft, Inc., Tulsa, OK, USA, 2016, STATISTICA-data analysis software system, version 13.1, www.statsoft.com (accessed on 14 April 2022)] software. Shapiro–Wilk normality test was used to examine the distribution of data. Statistical significance of differences in mRNA expression and age were determined by either with Mann–Whitney U test or Student *T*-test and data are presented as median [interquartile range] or mean ± SD depending on distribution. For age-dependent differences in the expression levels, 53 patients were divided into two subgroups: group 1: <60 years old (*n* = 32), group 2: ≥60 years old (*n* = 21). Percent contribution of the analyzed transporters was calculated using 2^−ΔCT^ values. Statistically significant differences (*p*-value) were assumed at *p* < 0.05.

## 3. Results

**Figure 1:**
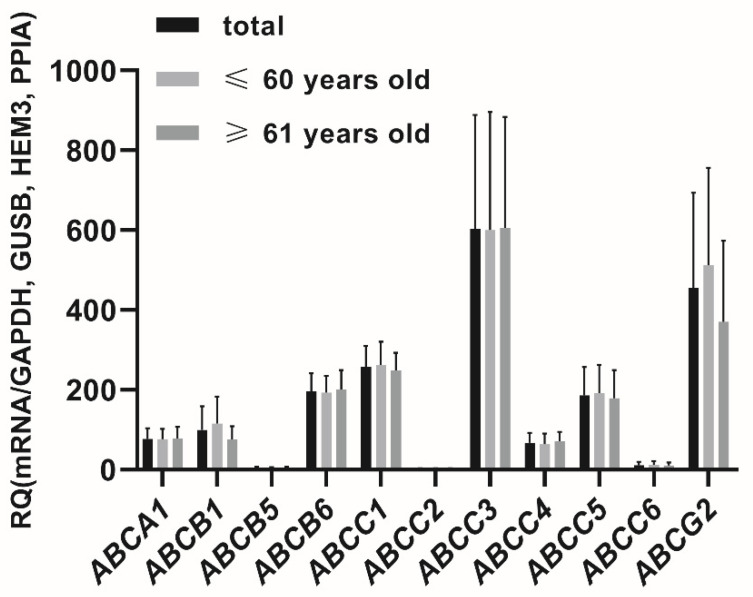
Expression of membrane transporters in the seminal vesicles. X: abbreviations of the examined ABC transporters superfamily. Y: relative quantification (RQ) 2^−ΔCT^ mean values (±SD).

**Figure 2:**
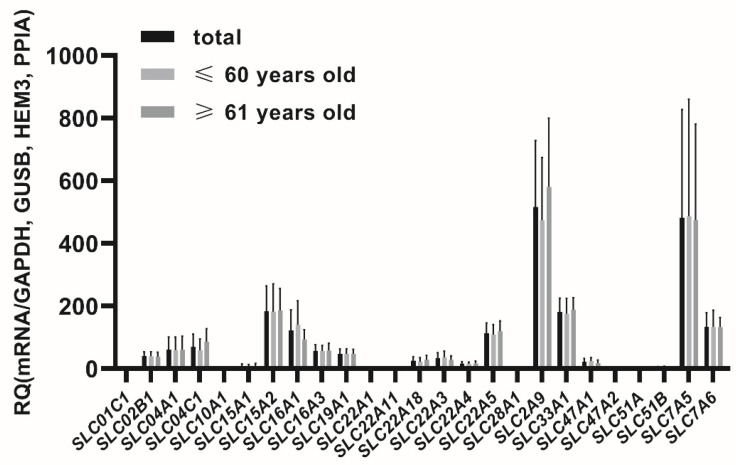
Expression of membrane transporters in the seminal vesicles. X: sbbreviations of the examined SLC superfamily. Y: relative quantification (RQ) 2^−ΔCT^ mean values (±SD).

In the seminal vesicles the following membrane transporters were defined at relevant expression levels (CT < 30 cycles, threshold 0.1), for ABC transporter superfamily: *ABCA1*, *ABCB1*, *ABCB5*, *ABCB6*, *ABCC1*, *ABCC2*, *ABCC3*, *ABCC4*, *ABCC5*, *ABCC6*, *ABCG2* and for SLC superfamily: *SLC01C1*, *SLC02B1*, *SLC04A1*, *SLC04C1*, *SLC10A1*, *SLC15A1*, *SLC15A2*, *SLC16A1*, *SLC16A3*, *SLC19A1*, *SLC22A1*, *SLC22A3*, *SLC22A11*, *SLC22A18*, *SLC22A4*, *SLC22A5*, *SLC28A1*, *SLC2A9*, *SLC33A1*, *SLC47A1*, *SLC47A2*, *SLC51A*, *SLC51B*, *SLC7A5*, *SLC7A6* (Figure 1 and Figure 2).

The percent contributions of the analyzed transporters in the seminal vesicles were defined as follows (2^−ΔCT^ values, sum of all analyzed transporters): *ABCC3* (14.76%), *SLC2A9* (12.65%), *SLC7A5* (11.81%), *ABCG2* (11.18%), *ABCC1* (6.30%), *ABCB6* (4.80%), *ABCC5* (4.57%), *SLC15A2* (4.50%), *SLC33A1* (4.43%), *SLC7A6* (3.25%), *SLC16A1* (2.98%), *SLC22A5* (2.77%), *ABCB1* (2.45%), *ABCA1* (1.89%), *SLC04C1* (1.71%), *ABCC4* (1.64%), *SLC04A1* (1.47%), *SLC16A3* (1.40%), *SLC19A1* (1.15%), *SLC02B1* (0.96%), *SLC22A3* (0.81%), *SLC22A18* (0.62%), *SLC47A1* (0.54%), *SLC22A4* (0.41%), *ABCC6* (0.28%), *SLC15A1* (0.16%), *SLC22A1* (0.11%), *SLC51B*, *ABCB5* (0.10%), *ABCC2* (0.07%), *SLC22A11*, *SLC47A2*, *SLC51A* (0.03%), *SLC01C1*, *SLC28A1*, *SLC10A1* (0.01%).

The study did not reveal relevant expressions of the following genes: *ABCG5*, *ABCG8* as well as *SLC01A2*, *SLC01B1*, *SLC01B3*, *SLC10A2*, *SLC22A2*, *SLC22A6*, *SLC22A7*, *SLC22A8*, *SLC22A9*, *SLC22A12*, *SLC28A2*.

**Figure 3:**
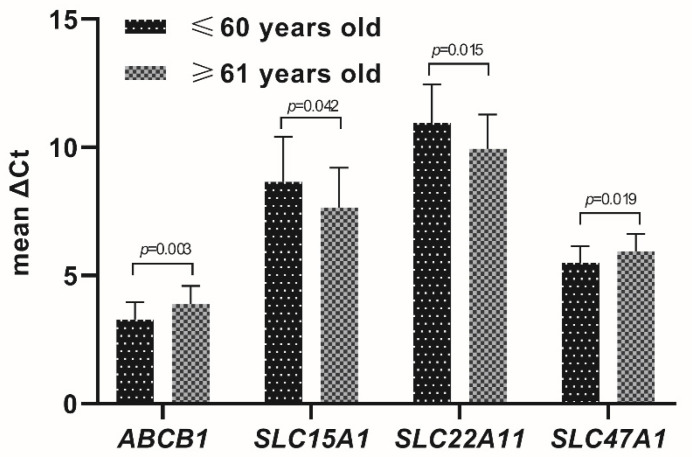
The selected transporters with significant age-dependent differences in the expression levels. X: Abbreviations of the examined ABC transporters and SLC superfamilies. Y: ΔCt mean values (±SD). Significance values: *ABCB1 p* = 0.003; *SLC15A1 p* = 0.042; *SLC22A11 p* = 0.015; *SCL47A1 p* = 0.019.

**Figure 4:**
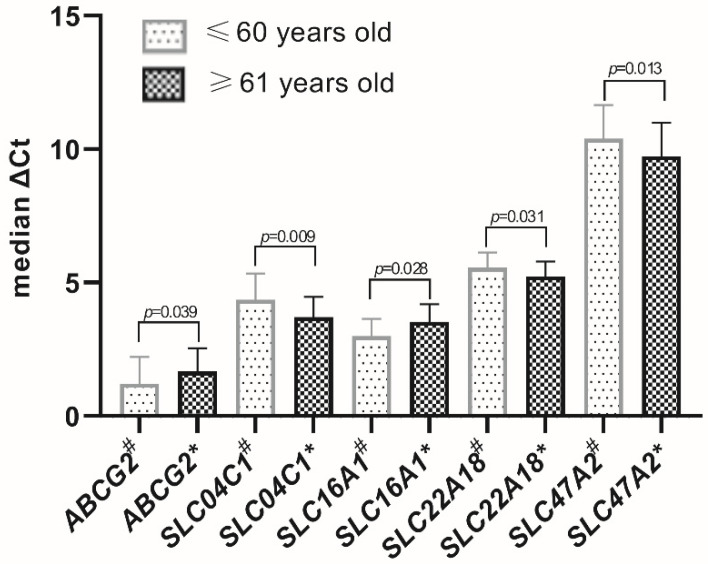
The selected transporters with significant age-dependent differences in the expression levels. X: abbreviations of the examined ABC transporters and SLC superfamilies; ^#^ ≤60 years old, * ≥61 years old. Y: ΔCt median values (IQR). Significance values: *ABCG2 p* = 0.039; *SLC04C1 p* = 0.009; *SLC16A1 p* = 0.028; *SLC22A18 p* = 0.031; *SLC47A2 p* = 0.013.

Age-dependent significant differences in the expression levels, stratifying the studied population as up to 60 (57.3 ± 2.78) and over 60 (67.8 ± 4.9) years of age, were evidenced for *ABCB1* and *ABCG2* as well as *SLC04C1*, *SLC15A1*, *SLC16A1*, *SLC22A11*, *SLC22A18*, *SLC47A1* and *SLC47A2* (Figure 3 and Figure 4). All other studied transporters did not demonstrate age-dependent changes.

**Figure 5:**
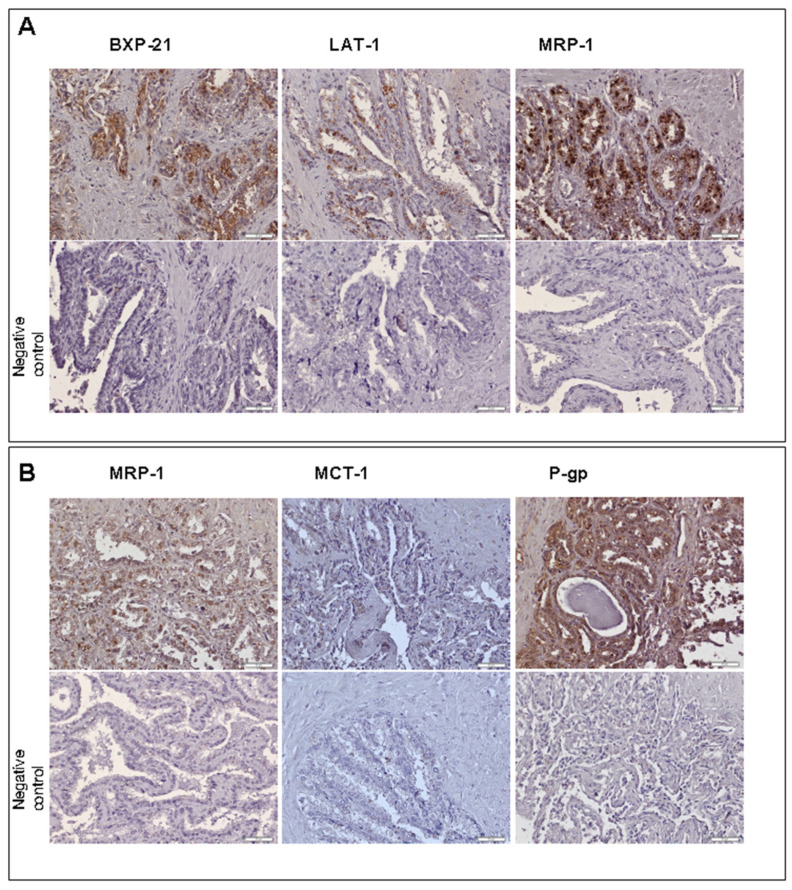
IHC expression and localization of transporters in seminal vesicles samples. Panel (**A**): upper line—BCRP, LAT1, MRP3 expressions in seminal vesicles tissue samples; lower line—negative control slides (without primary antibodies). Panel (**B**): upper line—MRP1, MCT1, P-gp expression in seminal vesicles tissue samples; lower line—negative controls. Original magnification ×200, scale bar 50 μm. Only representative images are included.

Immunohistochemistry analysis of the most abundantly expressed transporter genes (except for GLUT9 due to unavailability of a reliable antibody) revealed the presence of the transporter proteins of ABCG2 (*ABCG2*), MDR1/P-gp (*ABCB1*), MRP1 (*ABCC1*), MRP3 (*ABCC3*), MCT1 (*SLC16A1*) and LAT1 (*SLC7A1*) in human seminal vesicle cells. The staining revealed luminal localization of ABCG2, MDR1/P-gp, MRP1, MRP3, MCT1 and LAT1. The basolateral immunolocalization was defined for MDR1/P-gp (Figure 5).

## 4. Discussion

Membrane transporters and carriers provide efflux and influx activity, and thus regulate composition of intra- and extracellular fluids, affecting cell and organ functions. Each type of cell and organ is endowed with specific set of transporters and carriers. However, there is no information about their expression in the human seminal vesicles. The function of the vesicles, i.e., production of seminal plasma strongly suggest existence of transporter functions regulating the fluid composition. Likewise, in other barrier structures (e.g., blood–brain barrier or blood–testis barrier), membrane transporters also contribute to the mechanical barrier of tight junctions complementing the structure by active barrier functions [8].

The present study, for the first time, demonstrated relevant mRNA expression of 11 ABC transporters—*ABCA1*, *ABCB1*, *ABCB5*, *ABCB6*, *ABCC1*, *ABCC2*, *ABCC3*, *ABCC4*, *ABCC5*, *ABCC6*, *ABCG2*—and 25 SLC carriers and transporters—*SLC01C1*, *SLC02B1*, *SLC04A1*, *SLC04C1*, *SLC10A1*, *SLC15A1*, *SLC15A2*, *SLC16A1*, *SLC16A3*, *SLC19A1*, *SLC22A1*, *SLC22A3*, *SLC22A11*, *SLC22A18*, *SLC22A4*, *SLC22A5*, *SLC28A1*, *SLC2A9*, *SLC33A1*, *SLC47A1*, *SLC47A2*, *SLC51A*, *SLC51B*, *SLC7A5*, *SLC7A6*. The most abundant transporters and carriers were *ABCC3*, *SLC2A9*, *SLC7A5*, *ABCG2*, *ABCC1*, *ABCB6*, *ABCC5*, *SLC15A2*, *SLC33A1*, *SLC7A6* and *SLC16A1*. The antibody immunolocalization evidenced luminal localization of *ABCG2*, MDR1/P-gp, MRP1, MRP3, MCT1 and LAT1 as well as the basolateral immunolocalization of MDR1/P-gp in the seminal vesicles’ epithelia.

Among the transporters examined, the highest level of expression was demonstrated for the gene encoding MRP3 (*ABCC3*). MRP3 belongs to the subfamily of multiple drug resistance (MDR) proteins, but its exact biological characteristics is not entirely defined. This transporter has been shown to play a significant role in cell resistance to anticancer drugs (e.g., etoposide) [9]. In hepatocytes, MRP3 transports lipophilic anions such as bile acids and glucuronides, protecting cells from their toxicity [10]. The role of MRP3 in epithelial cells of the seminal vesicles is not defined, but it can be suggested that the transporter can be engaged in 3-α,17-β beta-androstanediol glucuronide [11] and in drug glucuronides transport into seminal fluid [12].

MRP1 (*ABCC1*) is a transporter highly expressed in the epithelial cells of seminal vesicles, which protein immunolocalization in the apical membrane was evidenced. As with MRP3, it belongs to MDR proteins, and was found to translocate neutral or positively charged, hydrophobic and amphipathic compounds (e.g., natural product antineoplastic agents—vincristine, doxorubicin) as well as organic anions (leukotriene C4, conjugates with glutathione, glucuronide, or sulfate). MRP1 has perhaps the widest variety of substrates of all the human ABC transporters [13]. The transporter operates in many tissues, and in the male reproductive tract it was found in basolateral membranes of Sertoli cells (being a part of the blood-testis barrier) and in Leydig cells. MRP1 is engaged in the transport of testosterone. In MRP1-deficient mice, decreased levels of testosterone were observed in serum and testes, despite unchanged expression and activity of steroid hormones-synthesizing enzymes in the testes and steroid-inactivating enzymes in the liver [14]. Moreover, mouse Leydig cells (MA-10) treated with an MRP1 inhibitor (MK-571) did not produce testosterone [15]. The role of MRP1 in the function of the seminal vesicle epithelium is not established, but it can be presumed that, as in other tissues and organs, the transporter can participate in maintaining the composition of seminal fluid and provide barrier functions.

BCRP (*ABCG2*) is the next ABC transporter localized in the apical membrane of the epithelial cells in seminal vesicles. Initially discovered as drug resistance transporter in cancer cells, breast cancer resistance protein (BCRP) is now defined in many cell types and tissues, especially those providing barrier functions (intestines, blood–brain barrier, placenta, mammary gland) [16]. The transporter translocates, apart from drugs, endogenous substrates, i.e., estrogens, porphyrins, bile acids, uric acid and riboflavin [17]. However, inability of BCRP to transport any of the androgen glucuronides, which is different from its highly active transport of several estrogen conjugates, has been documented [18]. In the human male reproductive tract, BCRP was evidenced in the blood–testis barrier. The presence of BCRP and other ABC efflux membrane transporters (P-gp, MRP1) prevents the accumulation of potentially harmful small molecules or lipophilic compounds in the testes, which shows its key role in protecting reproductive cells from circulating toxic substances [19].

P-gp, originally related to multiple drug resistance 1 (MDR1), was further defined as a membrane transporter expressed in many tissues and organs, playing a mainly protective role. In the male reproductive system, P-gp is one of the key components of the blood-testis barrier, where it operates in endothelial cells, and thus limits xeno-and endobiotics penetration. Sertoli and Leydig cells are also rich in P-gp [20]. It has been shown that mdr1-deficient mice more avidly accumulated P-gp substrates (e.g., ivermectin, vinblastine) in the testis as compared to controls [21]. It can be also assumed that P-gp in seminal vesicles’ epithelium can be engaged in the transport of testosterone and androstenedione, as was evidenced in in vitro models [22]. Thus, P-gp in seminal vesicles is likely involved in the protection of sperm cells against xenobiotics but also can affect the physiological microenvironment of the seminal plasma.

*SLC2A9* gene coding for glucose transporter 9 (GLUT9), was the highest (of the studied) expressed SLC transporters in the seminal vesicles. However, due to unavailability of the reliable antibodies, its immunolocalization and protein abundance were not studied. However, its high expression suggests that, as in other tissues and organs, it is mainly expressed in the kidneys, liver, and intestines, mediated urate transport [23]. A study in mice demonstrated GLUT9 presence in the plasma membrane of Leydig cells and sperm cells [24]. GLUT9 is mostly known as uric acid transporter. In the male reproductive tract uric acid contributes to maintain and enhance sperm motility and viability, and thus fertilizing ability. This contribution is produced mainly by neutralizing damaging effects of oxidizing (e.g., endogenous free radicals and exogenous toxins) and nitrating agents, and enhancing certain enzymes in spermatozoa. However, high levels of uric acid may induce deleterious effects to sperm function, at least in part, by reducing activity of enzymes in spermatozoa [25]. Apart from regulation of uric acid concentration in seminal plasma, GLUT9 function can also impact fructose concentrations as it was defined as a minor fructose transporter [26]. Fructose is produced by the seminal vesicles with some contribution from the ampulla of the ductus deferens, and based on the present study’s results it can be noted that GLUT9 may participate in its transport. Fructose is essential for spermatozoa metabolism and spermatozoa motility, being an energy source for spermatozoa [27]. Determination of seminal fructose concentration has been used in examination of obstructive azoospermia and inflammation of male accessory glands [28].

LAT1 (*SLC7A5*) is another ubiquitously expressed transporter, which carries a wide range of neutral amino acids, especially with large branched or aromatic side chains (e.g., tryptophan, phenylalanine, leucine, histidine). Experimental studies defined LAT1 expression in the male reproductive system, where the carrier was localized in the epididymis [29]. In the seminal vesicles and the epididymis, LAT1 can participate in amino acid transport, and takes part in the maintenance of a proper microenvironment for the process of sperm maturation, mobility and the ability to fertilize. Engel et al. revealed that all proteinogenic amino acids could be extracted from human seminal plasma (with the highest concentration of glutamine, glycine and serine), and it could be suggested that LAT1 contributes to their transport in the seminal vesicles. The seminal plasma content of most proteinogenic amino acids was significantly positively correlated with sperm concentration [30].

In the present study, the expression and immunolocalization of MCT1 (*SLC16A1*) were evidenced in the seminal vesicles. The transporter is ubiquitously expressed, and it takes lactate, pyruvate, butyrate, acetoacetate and β-hydroxybutyrate as predominant substrates [31]. In the testis, lactate is produced by Sertoli cells and transported to germline cells, and is a key metabolite for the normal occurrence of spermatogenesis. Deletion of MCT1 in Mct1 conditional knockout mice produced morphological changes in the seminiferous tubules and Sertoli cells, resulting in failure of spermatogenesis with depletion of germ cells and total absence of spermatoza [32]. Seminal plasma pyruvate and lactate can support sperm motility and mitochondrial function, and MCT1 in the seminal vesicles’ epithelia along with the activity lactate dehydrogenase may determine their availability. The enzyme activity deficiency in seminal plasma was correlated to infertility [33].

**Figure 6:**
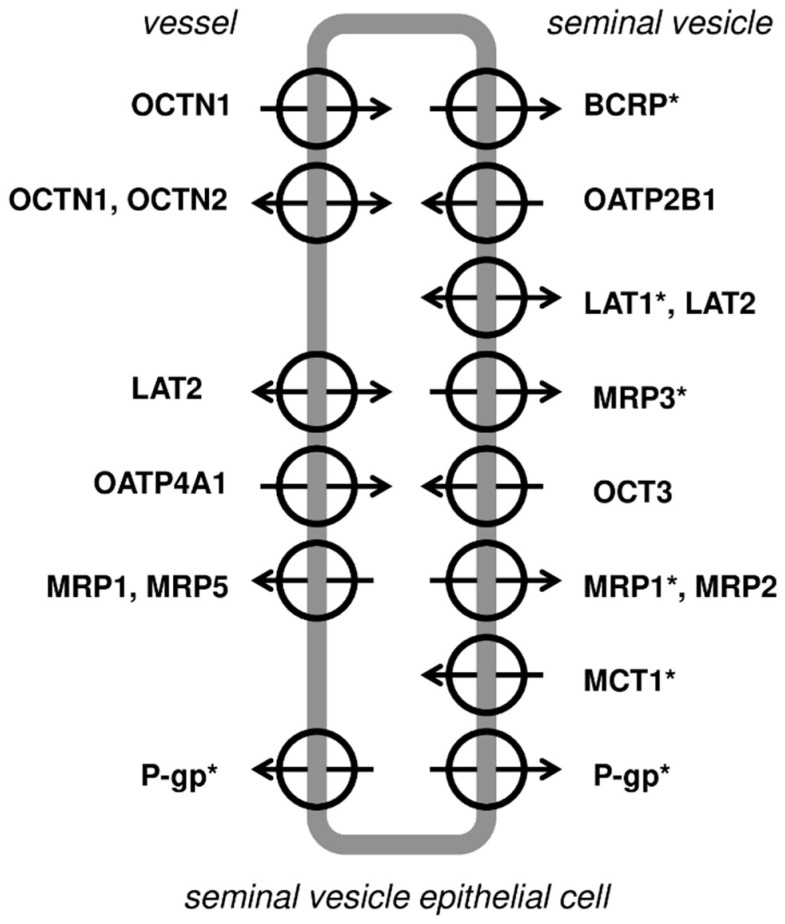
Membrane transporters in the human seminal vesicle epithelial cell (hypothetical distributions of the examined transporters). (*) Transporters immunolocalized in the present study. Putative localization of other transporters based on their functional role from literature data. BCRP—breast cancer resistance protein; LAT1, LAT2—L-Type Amino Acid Transporter 1, 2; MCT1—Monocarboxylate transporter 1; MRP1 MRP2, MRP3, MRP5—multidrug resistance-associated protein 1, 2, 3, 5; OATP2B1, OATP4A1—organic anion-transporting polypeptide 2B1, 4A1; OCT3—organic cation transporter 3; OCTN1, OCTN2—organic cation and carnitine transporter 1, 2; P-gp—P-glycoprotein.

The present study demonstrated immunolocaliaztion in the epithelium of the seminal vesicles both efflux (MRP1, MRP3, BCRP, P-gp) and influx carriers (LAT1 and MCT-1). Based on the current study observations, as well as the literature data, a hypothetical distribution of membrane transporters and carriers in the seminal vesicles can be outlined (see details in Figure 6).

In the current study, a significant upregulation of the expression levels (mRNA) of *ABCB1*, *ABCG2*, *SLC16A1* and *SLC47A1* as well as downregulation of *SLCO4A1*, *SLC15A1*, *SLC22A11*, *SLC22A18* and *SLC47A2* were found. The observed changes are related to the functional, physiological changes of the seminal vesicles’ epithelium [34]. The above results are in keeping with the existing literature showing age-dependent differences in the expression of genes encoding membrane transporters and carriers in the lung, brain, heart or gastrointestinal tract [35].

The present study for the first time provides a broad expression characteristics of membrane transporters and carriers in human seminal vesicles. The information presented may enable better understanding of pathophysiological processes in the organ. Active and passive transport across the membranes can regulate seminal fluid composition (both physiological and deleterious/toxic factors content), which in turn can affect sperm quality and male fertility potential. From other organ studies it is known that various factors modulate transporter expression and function, and likewise in seminal vesicles, an inflammation process, drugs, toxins can affect transporters and carriers. Interactions between membrane transporters and microbiome is one of the emerging areas of investigation. Observations from the gastrointestinal tract suggest that microbiome products, e.g., short-chain fatty acids, can interact with enterocyte transport functions, and after absorption in remote organs (the blood–brain barrier or kidneys). Experimental findings provide evidence that diet or antibiotics can modify seminal fluid microbiome, and thus the microbiome can be another mediator of interactions between environmental factors and membrane transporter functions [36,37].

The results of the present study have some limitations, e.g., the analysis of samples from prostate cancer subjects, which might affect the picture of the examined transporters in seminal vesicles. Likewise, relatively elderly samples were analyzed, and the panel of membrane transporters and carriers was assessed only in subjects over 50 years of age, which may vary from younger individuals. Therefore, the results from the present study should be analyzed with some restrictions, and more studies are needed to define the role of membrane transport in physiology and pathological states of human seminal vesicles.

## 5. Conclusions

The study provides, for the first time, information about the expression of membrane transporters and carriers in the seminal vesicles. The localization of transporters can in part explain the contribution of the seminal vesicles to the composition of seminal plasma, and confirm the secretory ability of the epithelium. Expression of the ATP-binding cassette transporters, which are expressed not only in secretory epithelia, but also in many barrier organs (e.g., blood–brain or blood–testis barrier), suggests that the seminal vesicle epithelium may also provide protective role, limiting penetration of toxic molecules to seminal plasma.

## Data Availability

Not applicable.

## Supplementary Materials

The following supporting information can be downloaded at: https://www.mdpi.com/article/10.3390/jcm11082213/s1, Table S1: Detailed characteristics of study objects; Table S2: TaqMan Gene Expression Assays.
