# Supplementary material for: Membrane Transporters and Carriers in Human Seminal Vesicles
Source: J Clin Med. 2022 Apr 15;11(8):2213. doi: 10.3390/jcm11082213 (PMC9029209; doi:10.3390/jcm11082213)
Supplement: Supplementary file 1 [file jcm-11-02213-s001.zip › jcm-1613082-supplementary.pdf]

Membrane transporters and carriers in human seminal vesicles

Damian Malinowski, Paweł Grzegółkowski, Katarzyna Piotrowska, Marcin Słojewski and Marek Droździk

Supplementary Table S1. Detailed characteristics of study objects.

sample number	AGE [years]	WEIGHT [kg]	HEIGHT [cm]	BMI	ECOG STATUS [0-5]	MARITAL STATUS	PRIOR SEXUAL ACTIVITY	PSA [ng/m]	NAC / RT / HT	COMORBIDITIES	MEDICATION
1	63	104	174	34	0	MARRIED	ACTIVE	7,00	A-RT (dose 60 Gy)	HYPERTENSION, GOUT	NEBIVOLOL, ALLOPURINOLUM
2	69	80	170	28	1	MARRIED	ACTIVE	19,80	A-RT (dose 60 Gy)	HYPERTENSION	VALSARTAN, HYDROCHLOROTHIAZIDE
3	78	78	177	25	1	WIDOWER	NON-ACTIVE	7,00	NONE	NONE	NONE
4	61	77	179	24	0	MARRIED	ACTIVE	8,90	NONE	HYPERTENSION	NEBIVOLOL
5	67	85	177	27	0	MARRIED	ACTIVE	7,00	NONE	HYPERTENSION	NONE
6	68	86	179	27	0	MARRIED	ACTIVE	8,00	HT (goserelinum)	HYPERTENSION, GOUT	ALLOPURINOLUM
7	65	76	176	25	0	MARRIED	ACTIVE	8,00	NONE	HYPERTENSION	NONE
8	71	84	170	29	1	MARRIED	ACTIVE	9,00	NONE	HYPERTENSION	INDAPAMIDE
9	62	89	176	29	0	MARRIED	ACTIVE	16,00	NONE	NONE	NONE

10	62	80	179	25	1	MARRIED	ACTIVE	7,00	NONE	NONE	NONE
11	63	92	182	28	0	MARRIED	ACTIVE	8,00	NONE	HYPERTENSION, HYPOTHYROIDSM	LEVOTHYROXINE
12	59	91	176	29	1	MARRIED	NON-ACTIVE	8,90	NONE	HYPERTENSION, HYPOTHYROIDSM	INDAPAMIDE, CHLORTALIDONE
13	55	105	182	32	0	MARRIED	NON-ACTIVE	13,00	NONE	NONE	NONE
14	63	98	176	32	0	MARRIED	ACTIVE	9,00	NONE	HYPERTENSION	INDAPAMIDE
15	60	80	176	26	1	MARRIED	ACTIVE	28,00	H-RT (goserelinum + dose 66 Gy)	NONE	NONE
16	60	78	175	25	1	MARRIED	ACTIVE	9,80	NONE	HYPERTENSION	INDAPAMIDE
17	58	85	185	25	0	MARRIED	ACTIVE	9,20	NONE	NONE	NONE
18	75	83	175	27	1	MARRIED	ACTIVE	7,70	NONE	NONE	NONE
19	60	90	179	28	0	MARRIED	ACTIVE	16,00	H-RT (goserelinum + dose 66 Gy)	NONE	NONE
20	59	78	175	25	0	MARRIED	ACTIVE	6,80	NONE	NONE	NONE
21	58	82	180	25	0	MARRIED	ACTIVE	4,00	NONE	HYPERTENSION	NEBIVOLOL, INDAPAMIDE, ATORVASTATIN

22	53	78	169	27	0	MARRIED	ACTIVE	17,30	H-RT (goserelinum + dose 60 Gy)	NONE	NONE
23	53	122	176	39	0	MARRIED	NON-ACTIVE	13,50	NONE	HYPERTENSION, DIABETES TYPE 2	AMILORIDE, ALLOPURINOLUM, GLUCOPHAGE, BISOPROLOL, RAMIPRIL
24	58	90	180	28	0	MARRIED	ACTIVE	5,20	NONE	HYPERTENSION	VALSARTAN, ENALAPRIL
25	57	97	174	32	0	MARRIED	ACTIVE	11,00	NONE	NONE	NONE
26	55	87	180	27	0	MARRIED	ACTIVE	6,30	NONE	HYPERTENSION, HYPOTHYROIDSM	LEVOTHYROXINE Concor
27	59	97	174	32	0	MARRIED	ACTIVE	5,20	RT (dose 60 Gy)	HYPERTENSION	RAMIPRIL
28	59	73	170	25	0	WIDOWER	ACTIVE	16,00	NONE	HYPERTENSION	VALSARTAN, HYDROCHLOROTHIAZIDE
29	57	80	184	24	0	MARRIED	ACTIVE	8,38	NONE	NONE	NONE
30	60	90	179	28	1	MARRIED	ACTIVE	16,40	H-RT (goserelinum + dose 66 Gy)	HYPERTENSION, DIABETES TYPE 2	METFORMIN, FENOFIBRATE, LACIDIPINE, BISOPROLOL, QUINAPRIL
31	60	77	169	27	0	MARRIED	ACTIVE	5,00	NONE	HYPERTENSION	PERINDOPRIL, AMLODIPINE
32	54	100	182	30	0	MARRIED	ACTIVE	19,00	H-RT (goserelin	HYPERTENSION	NONE

									um + dose 60 Gy)		
33	73	80	173	27	1	MARRIED	ACTIVE	7,40	NONE	HYPERTENSION, DIABETES TYPE 2	METFORMIN, GLICLAZIDE, NEBIVOLOL, ACETYLSALICYLIC ACID, DOXAZOSIN, SIMVASTATIN
34	60	104	170	36	0	MARRIED	ACTIVE	5,50	A-RT (goserelin um + dose 66 Gy)	HYPOTHYROIDSM	LEVOTHYROXINE
35	71	82	179	26	1	MARRIED	NON-ACTIVE	9,00	NONE	DIABETES TYPE 2	GLUCOPHAGE
36	60	77	174	25	0	MARRIED	ACTIVE	7,00	NONE	DIABETES TYPE 2, RHEUMATOID ARTHRITIS	LOSARTAN, FENOFIBRATE, DILTIAZEM, METFORMIN
37	70	82	179	26	1	MARRIED	ACTIVE	6,47	NONE	HYPERTENSION	VALSARTAN, HYDROCHLOROTHIA ZIDE
38	57	74	182	22	0	MARRIED	ACTIVE	5,22	NONE	NONE	NONE
39	58	76	177	24	0	MARRIED	ACTIVE	9,80	NONE	NONE	NONE
40	73	80	173	27	1	MARRIED	NON-ACTIVE	4,70	NONE	HYPERTENSION, DIABETES TYPE 2	METFORMIN, BIOPRAZOLUM
41	48	75	178	24	0	MARRIED	ACTIVE	6,60	NONE	HYPERTENSION	RAMIPRIL
42	60	82	180	25	0	MARRIED	ACTIVE	6,50	NONE	HYPERTENSION	SOTALOL

43	55	80	172	27	0	MARRIED	ACTIVE	9,00	NONE	HYPOTHYROIDSM	LEVOTHYROXINE
44	57	89	174	29	0	MARRIED	ACTIVE	6,38	NONE	NONE	NONE
45	57	82	172	28	0	MARRIED	ACTIVE	7,40	A-RT (dose 60 Gy)	HYPERTENSION	TORASEMIDE, QUINAPRIL
46	64	79	178	25	0	MARRIED	ACTIVE	13,64	NONE	HYPERTENSION	SOTALOL
47	71	68	165	25	1	MARRIED	ACTIVE	4,24	NONE	HYPERTENSION, ISCHEMIC HEART DISEASE	ACETYLSALICYLIC ACID, AMLODIPINE, RAMIPRIL, ATORVASTATIN
48	60	83	180	26	0	MARRIED	ACTIVE	9,00	NONE	HYPERTENSION	VALSARTAN INDAPAMIDE TAMSULOSIN
49	60	80	163	30	0	MARRIED	ACTIVE	10,40	NONE	HYPERTENSION, DIABETES TYPE 2	CELIPROLOL, ATORVASTATIN, ACETYLSALICYLIC ACID, INDAPAMIDE, TAMSULOSIN, METFORMIN
50	73	85	170	29	1	MARRIED	ACTIVE	12,50	NONE	HYPERTENSION, ISCHEMIC HEART DISEASE, GLAUCOMA	NEBIVOLOL, VALSARTAN, AMLODIPINE, ACETYLSALICYLIC ACID
51	54	83	181	25	0	MARRIED	ACTIVE	9,33	NONE	HYPERTENSION, DIABETES TYPE 2	TAMSULOSIN, PERINDOPRIL, NEBIVOLOL, ATORVASTEROL
52	71	78	177	25	1	MARRIED	ACTIVE	5,07	NONE	HYPERTENSION, DIABETES TYPE 2	TELMISARTAN, POLFENON, BISOPROLOL, METFORMIN,

											BETAHISTINE, TAMSULOSIN
53	56	86	179	27	0	MARRIED	ACTIVE	5,70	NONE	NONE	NONE

ECOG—Eastern Cooperative Oncology Group Performance Status (ECOG performance status: 0—Fully active, able to carry on all pre-disease performance without restriction; 1—Restricted in physically strenuous activity but ambulatory and able to carry out work of a light or sedentary nature, e.g., light house work, office work; 2—Ambulatory and capable of all selfcare but unable to carry out any work activities; up and about more than 50% of waking hours; 3—Capable of only limited selfcare; confined to bed or chair more than 50% of waking hours; 4—Completely disabled; cannot carry on any selfcare; totally confined to bed or chair; 5—Dead); NAC—Neoadjuvant Chemotherapy; RT—Radiation Therapy; HT—Hormonal Therapy; A-HT—Adjuvant Hormonal Therapy; A-RT—Adjuvant Radiation Therapy; A-HRT—Adjuvant Radiohormonotherapy

Supplementary Table S2. TaqMan Gene Expression Assays

ABC transporters		SLC transporters		Housekeeping genes	
**Gene symbol**	**TaqMan assay ID**	**Gene symbol**	**TaqMan assay ID**	**Gene symbol**	**TaqMan assay ID**
ABCA1	Hs00442663_m1	SLC01A2	Hs00366488_m1	GAPDH	Hs99999905_m1
ABCB1	Hs00184500_m1	SLC01B1	Hs00272374_m1	GUSB	Hs99999908_m1
ABCB5	Hs02889060_m1	SLC01B3	Hs00351987_m1	HEM3	Hs00609297_m1
ABCB6	Hs00180568_m1	SLC01C1	Hs00213714_m1	PPIA	Hs99999904_m1
ABCC1	Hs01561502_m1	SLC02B1	Hs01030353_m1		
ABCC2	Hs00166123_m1	SLC04A1	Hs00983988_m1		
ABCC3	Hs00978473_m1	SLC04C1	Hs00698884_m1		
ABCC4	Hs00988717_m1	SLC10A1	Hs00161820_m1		
ABCC5	Hs00981089_m1	SLC10A2	Hs01001557_m1		
ABCC6	Hs00184566_m1	SLC15A1	Hs00192639_m1		
ABCG2	Hs01053790_m1	SLC15A2	Hs01113665_m1		
ABCG5	Hs00223686_m1	SLC16A1	Hs01560299_m1		
ABCG8	Hs00223690_m1	SLC16A3	Hs00358829_m1		
		SLC19A1	Hs00953344_m1		
		SLC22A1	Hs00427552_m1		
		SLC22A11	Hs00945829_m1		
		SLC22A12	Hs00375985_m1		
		SLC22A18	Hs00180039_m1		
		SLC22A2	Hs01010726_m1		
		SLC22A3	Hs00222691_m1		
		SLC22A4	Hs01548718_m1		
		SLC22A5	Hs00929869_m1		
		SLC22A6	Hs00537914_m1		
		SLC22A7	Hs00198527_m1		
		SLC22A8	Hs00188599_m1		
		SLC22A9	Hs00375768_m1		
		SLC28A1	Hs00984403_m1		

SLC28A2	Hs00188407_m1
SLC2A9	Hs00417125_m1
SLC33A1	Hs00270469_m1
SLC47A1	Hs00217320_m1
SLC47A2	Hs00945650_m1
SLC51A	Hs00380895_m1
SLC51B	Hs01057182_m1
SLC7A5	Hs01001183_m1
SLC7A6	Hs00187727_m1
